# Supplementary material for: Metabolic signature of the pathogenic 22q11.2 deletion identifies carriers and provides insight into systemic dysregulation
Source: Transl Psychiatry. 2023 Dec 14;13:391. doi: 10.1038/s41398-023-02697-8 (PMC10721888; doi:10.1038/s41398-023-02697-8)
Supplement: Supplementary file 1 — Supplementary Material [file 41398_2023_2697_MOESM1_ESM.pdf]

Metabolic signature of the pathogenic 22q11.2 deletion identifies carriers and provides insight into systemic dysregulation
Supplementary Information

Julie Courraud[§] (1,2,3,4), Francesco Russo[§] (1), Gonçalo Espregueira Themudo (2,5,6,7), Susan Svane Laursen (1,2), Andrés Ingason (2, 8), David M. Hougaard (1,2), Arieh S. Cohen (1,2), Thomas Werge* (2,5,9,10), Madeleine Ernst* (1,2)

Affiliations

(1) Section for Clinical Mass Spectrometry, Danish Center for Neonatal Screening, Department of Congenital Disorders, Statens Serum Institut, Artillerivej 5, DK-2300 Copenhagen S, Denmark

(2) iPSYCH, The Lundbeck Foundation Initiative for Integrative Psychiatric Research, Copenhagen, Denmark

(3) Laboratory of Analytical Chemistry, Department of Chemistry, National and Kapodistrian University of Athens, Panepistimiopolis Zografou, 15771, Athens, Greece

(4) Department of Clinical Therapeutics, School of Medicine, National and Kapodistrian University of Athens, Alexandra Hospital, Leof. Vasilissis Sofias 80, Athens 11528, Greece

(5) Institute of Biological Psychiatry, Copenhagen University Hospital, Copenhagen Mental Health Services, Kristineberg 3, DK-2100 Copenhagen Ø, Denmark

(6) CIIMAR, Interdisciplinary Centre of Marine and Environmental Research, University of Porto, Terminal de Cruzeiros do Porto de Leixões, Avenida General Norton de Matos, S/N, 4450-208 Matosinhos, Portugal

(7) Centre for Ecology, Evolution and Environmental Changes (CE3C), Faculdade de Ciências da Universidade de Lisboa, Campo Grande, 1749-016 Lisboa, Portugal

(8) Institute of Biological Psychiatry, Mental Health Center Sankt Hans, DK-4000 Roskilde, Denmark

(9) Department of Clinical Sciences, Faculty of Health, University of Copenhagen, Blegdamsvej 3, DK-2200 København N, Denmark

(10) GLOBE Institute, LF Center for GeoGenetics, Faculty of Health, University of Copenhagen, Oester Voldgade 5-7, 1350 Copenhagen K, Denmark

§ These authors contributed equally to this work

* Correspondence should be addressed to Madeleine Ernst (maet@ssi.dk) regarding mass spectrometry and metabolomics data analysis and interpretation and Thomas Werge (thomas.werge@regionh.dk) regarding 22q11.2 and clinical data interpretation

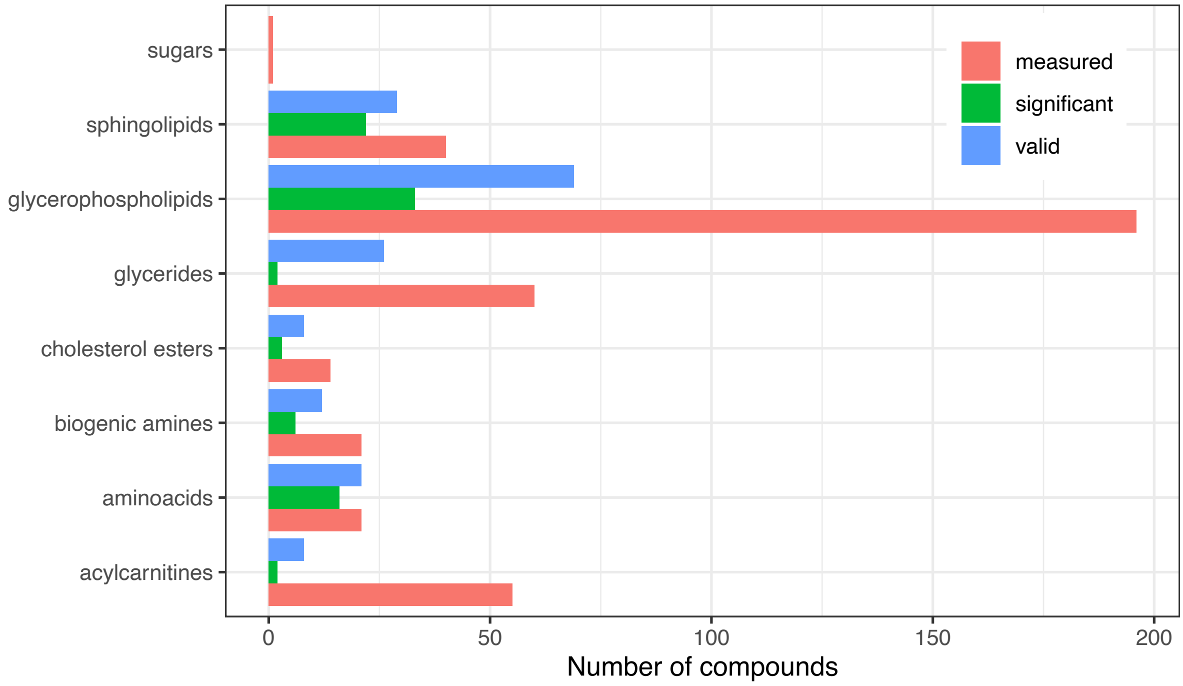

Supplementary Figure 1. Number of measured, valid, and significantly differentially abundant compounds across 22q11.2 carriers and controls stratified per compound class. Barplots showing the total number of compounds measured (n = 408), the total number of compounds, which passed quality control (n = 173), and the total number of compounds, which were differentially abundant (FDR-adjusted p-value < 0.05) across 22q11.2 carriers and controls using a paired Wilcoxon signed-rank test (n = 84).

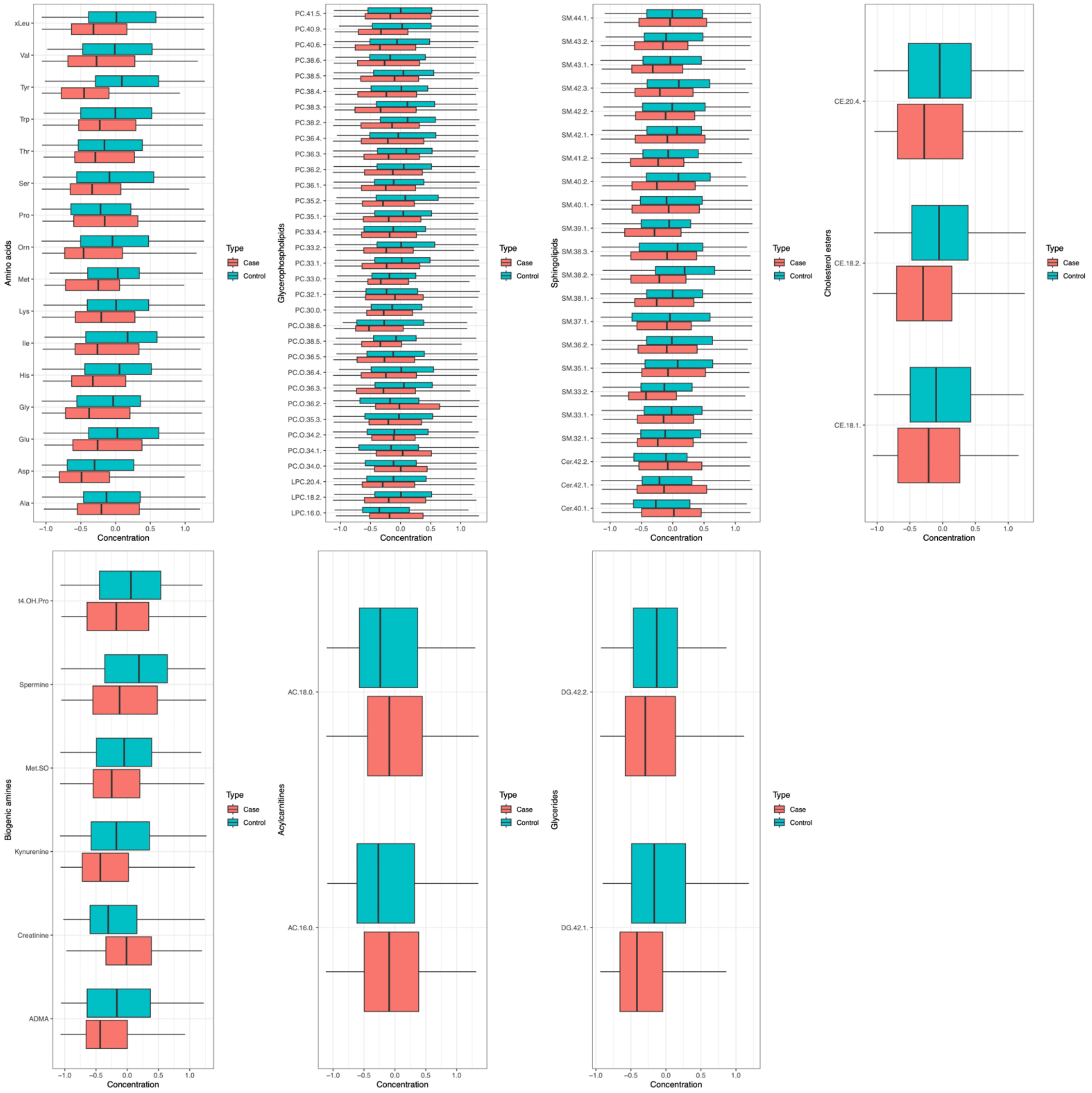

Supplementary Figure 2. Differentially abundant metabolites between 22q11.2 carriers and controls stratified by compound class. Boxplots show differentially abundant metabolites (FDR-adjusted p-value < 0.05, n=84) between 22q11.2 carriers and controls using a paired Wilcoxon signed-rank test and grouped by compound classes.
