# Supplementary material for: Metabolic signature of the pathogenic 22q11.2 deletion identifies carriers and provides insight into systemic dysregulation
Source: Transl Psychiatry. 2023 Dec 14;13:391. doi: 10.1038/s41398-023-02697-8 (PMC10721888; doi:10.1038/s41398-023-02697-8)
Supplement: Supplementary file 2 — Supplementary Data 1 [file 41398_2023_2697_MOESM2_ESM.pdf]

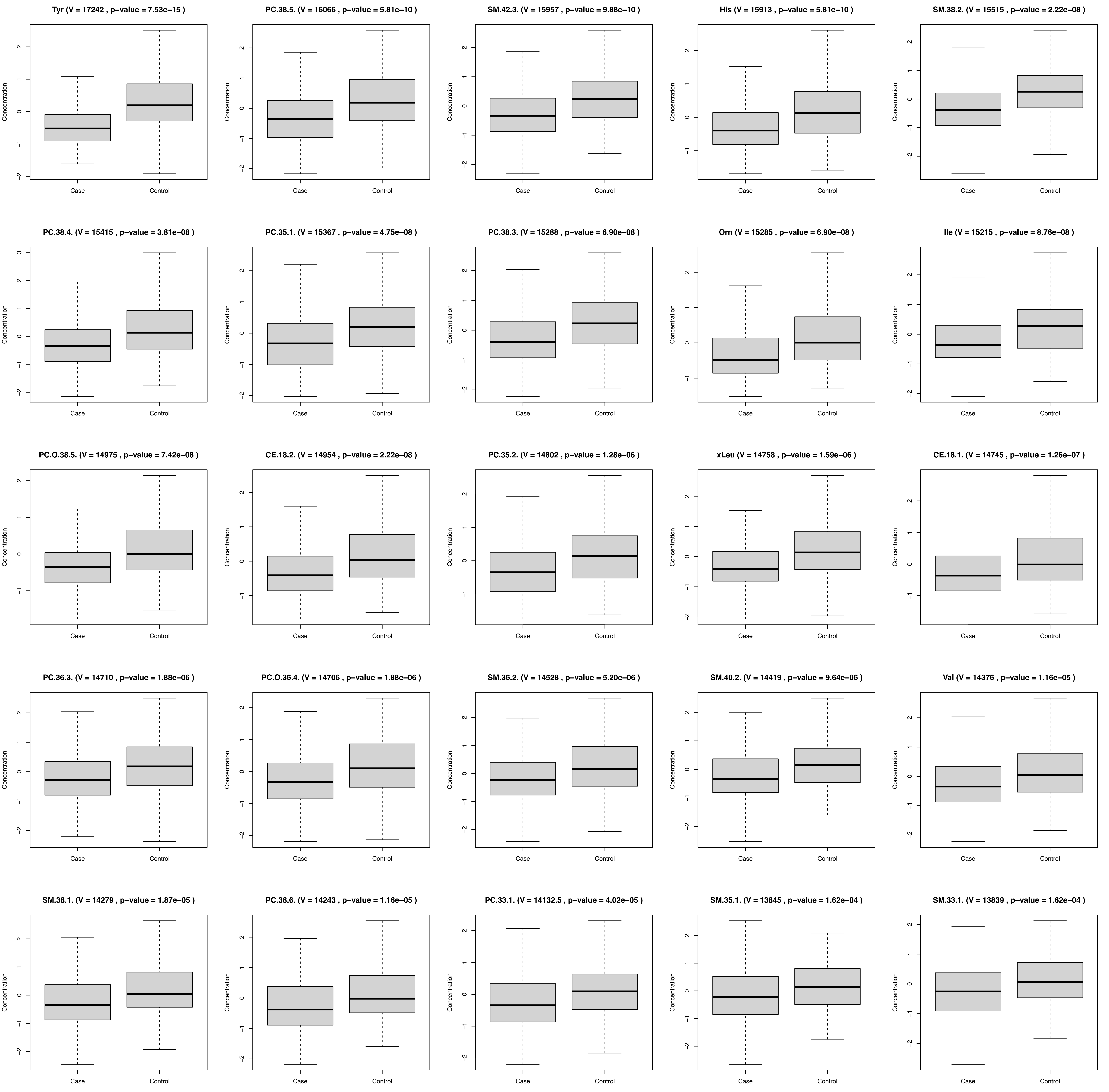
Tyr (V = 17242 , p−value = 7.53e−15)
PC.38.5. (V = 16066 , p−value = 5.81e−10)
SM.42.3. (V = 15957 , p−value = 9.88e−10)
His (V = 15913 , p−value = 5.81e−10)
SM.38.2. (V = 15515 , p−value = 2.22e−08)
PC.38.4. (V = 15415 , p−value = 3.81e−08)
PC.35.1. (V = 15367 , p−value = 4.75e−08)
PC.38.3. (V = 15288 , p−value = 6.90e−08)
Orn (V = 15285 , p−value = 6.90e−08)
Ile (V = 15215 , p−value = 8.76e−08)
PC.O.38.5. (V = 14975 , p−value = 7.42e−08)
CE.18.2. (V = 14954 , p−value = 2.22e−08)
PC.35.2. (V = 14802 , p−value = 1.28e−06)
xLeu (V = 14758 , p−value = 1.59e−06)
CE.18.1. (V = 14745 , p−value = 1.26e−07)
PC.36.3. (V = 14710 , p−value = 1.88e−06)
PC.O.36.4. (V = 14706 , p−value = 1.88e−06)
SM.36.2. (V = 14528 , p−value = 5.20e−06)
SM.40.2. (V = 14419 , p−value = 9.64e−06)
Val (V = 14376 , p−value = 1.16e−05)
SM.38.1. (V = 14279 , p−value = 1.87e−05)
PC.38.6. (V = 14243 , p−value = 1.16e−05)
PC.33.1. (V = 14132.5 , p−value = 4.02e−05)
SM.35.1. (V = 13845 , p−value = 1.62e−04)
SM.33.1. (V = 13839 , p−value = 1.62e−04)
Concentration
Case
Control

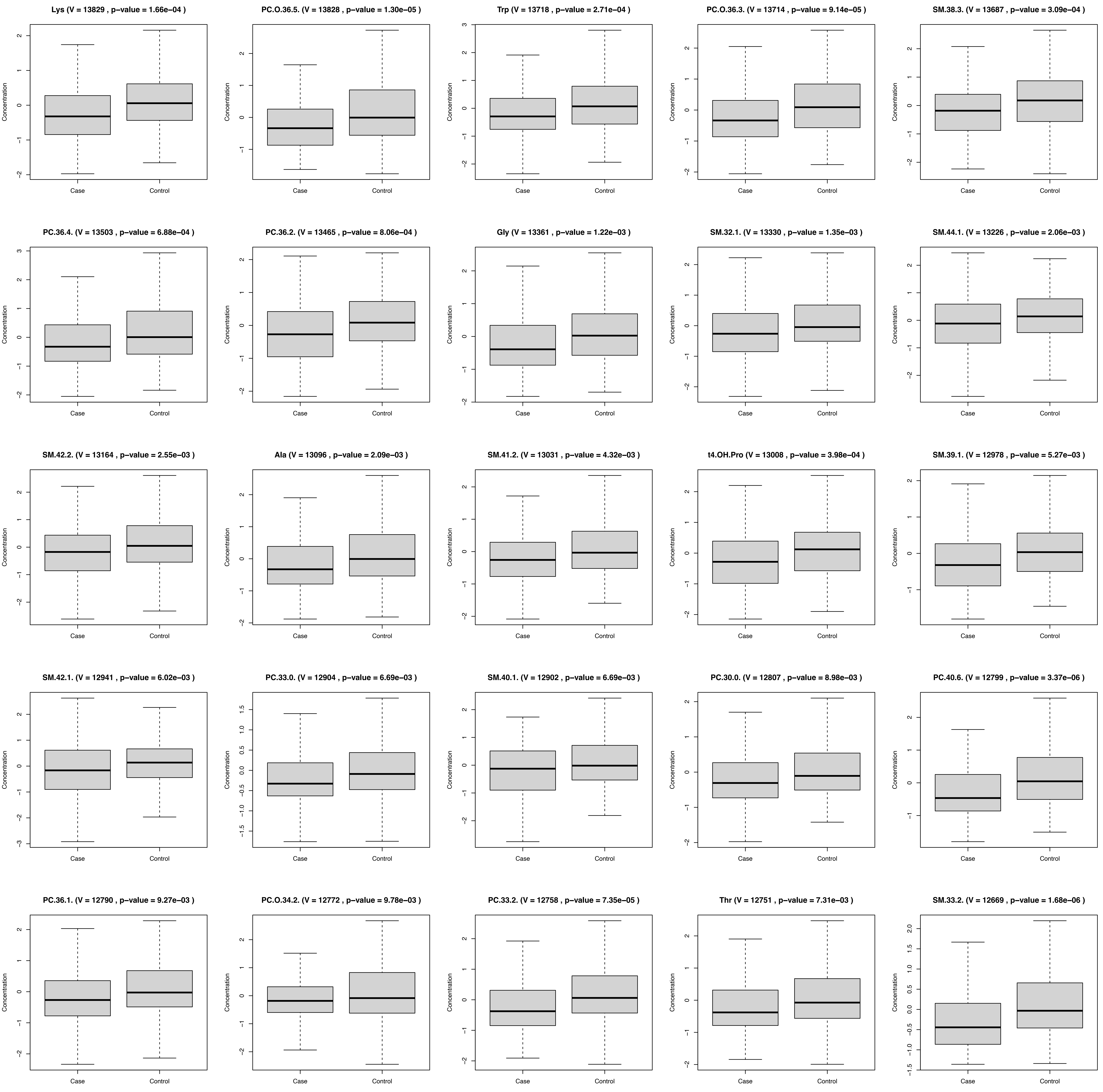

Lys (V = 13829 , p−value = 1.66e−04)
PC.O.36.5. (V = 13828 , p−value = 1.30e−05)
Trp (V = 13718 , p−value = 2.71e−04)
PC.O.36.3. (V = 13714 , p−value = 9.14e−05)
SM.38.3. (V = 13687 , p−value = 3.09e−04)
PC.36.4. (V = 13503 , p−value = 6.88e−04)
PC.36.2. (V = 13465 , p−value = 8.06e−04)
Gly (V = 13361 , p−value = 1.22e−03)
SM.32.1. (V = 13330 , p−value = 1.35e−03)
SM.44.1. (V = 13226 , p−value = 2.06e−03)
SM.42.2. (V = 13164 , p−value = 2.55e−03)
Ala (V = 13096 , p−value = 2.09e−03)
SM.41.2. (V = 13031 , p−value = 4.32e−03)
t4.OH.Pro (V = 13008 , p−value = 3.98e−04)
SM.39.1. (V = 12978 , p−value = 5.27e−03)
SM.42.1. (V = 12941 , p−value = 6.02e−03)
PC.33.0. (V = 12904 , p−value = 6.69e−03)
SM.40.1. (V = 12902 , p−value = 6.69e−03)
PC.30.0. (V = 12807 , p−value = 8.98e−03)
PC.40.6. (V = 12799 , p−value = 3.37e−06)
PC.36.1. (V = 12790 , p−value = 9.27e−03)
PC.O.34.2. (V = 12772 , p−value = 9.78e−03)
PC.33.2. (V = 12758 , p−value = 7.35e−05)
Thr (V = 12751 , p−value = 7.31e−03)
SM.33.2. (V = 12669 , p−value = 1.68e−06)
Concentration
Case
Control

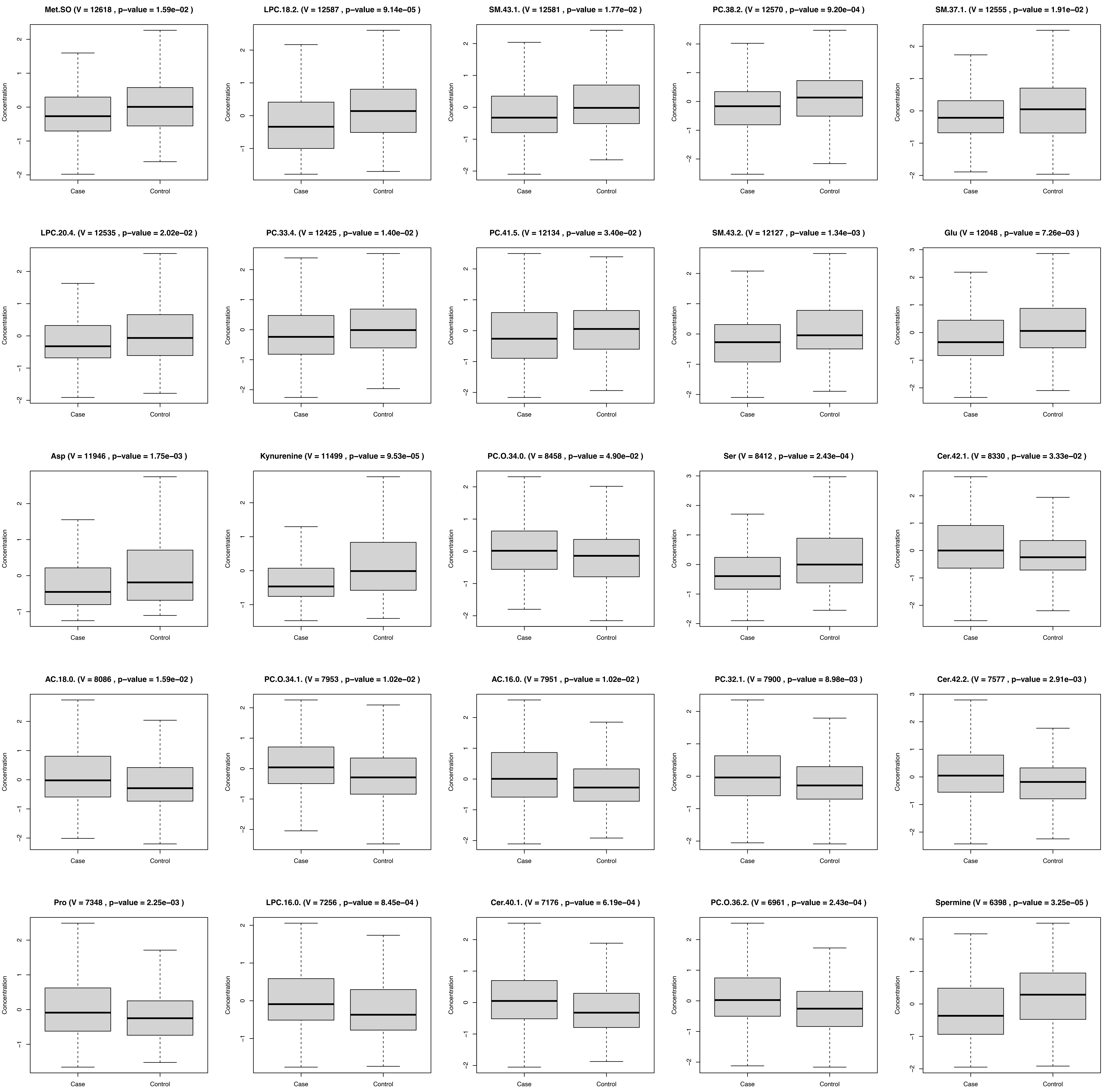

Met.SO (V = 12618 , p−value = 1.59e−02)
LPC.18.2. (V = 12587 , p−value = 9.14e−05)
SM.43.1. (V = 12581 , p−value = 1.77e−02)
PC.38.2. (V = 12570 , p−value = 9.20e−04)
SM.37.1. (V = 12555 , p−value = 1.91e−02)
LPC.20.4. (V = 12535 , p−value = 2.02e−02)
PC.33.4. (V = 12425 , p−value = 1.40e−02)
PC.41.5. (V = 12134 , p−value = 3.40e−02)
SM.43.2. (V = 12127 , p−value = 1.34e−03)
Glu (V = 12048 , p−value = 7.26e−03)
Asp (V = 11946 , p−value = 1.75e−03)
Kynurenine (V = 11499 , p−value = 9.53e−05)
PC.O.34.0. (V = 8458 , p−value = 4.90e−02)
Ser (V = 8412 , p−value = 2.43e−04)
Cer.42.1. (V = 8330 , p−value = 3.33e−02)
AC.18.0. (V = 8086 , p−value = 1.59e−02)
PC.O.34.1. (V = 7953 , p−value = 1.02e−02)
AC.16.0. (V = 7951 , p−value = 1.02e−02)
PC.32.1. (V = 7900 , p−value = 8.98e−03)
Cer.42.2. (V = 7577 , p−value = 2.91e−03)
Pro (V = 7348 , p−value = 2.25e−03)
LPC.16.0. (V = 7256 , p−value = 8.45e−04)
Cer.40.1. (V = 7176 , p−value = 6.19e−04)
PC.O.36.2. (V = 6961 , p−value = 2.43e−04)
Spermine (V = 6398 , p−value = 3.25e−05)
Concentration
Case
Control

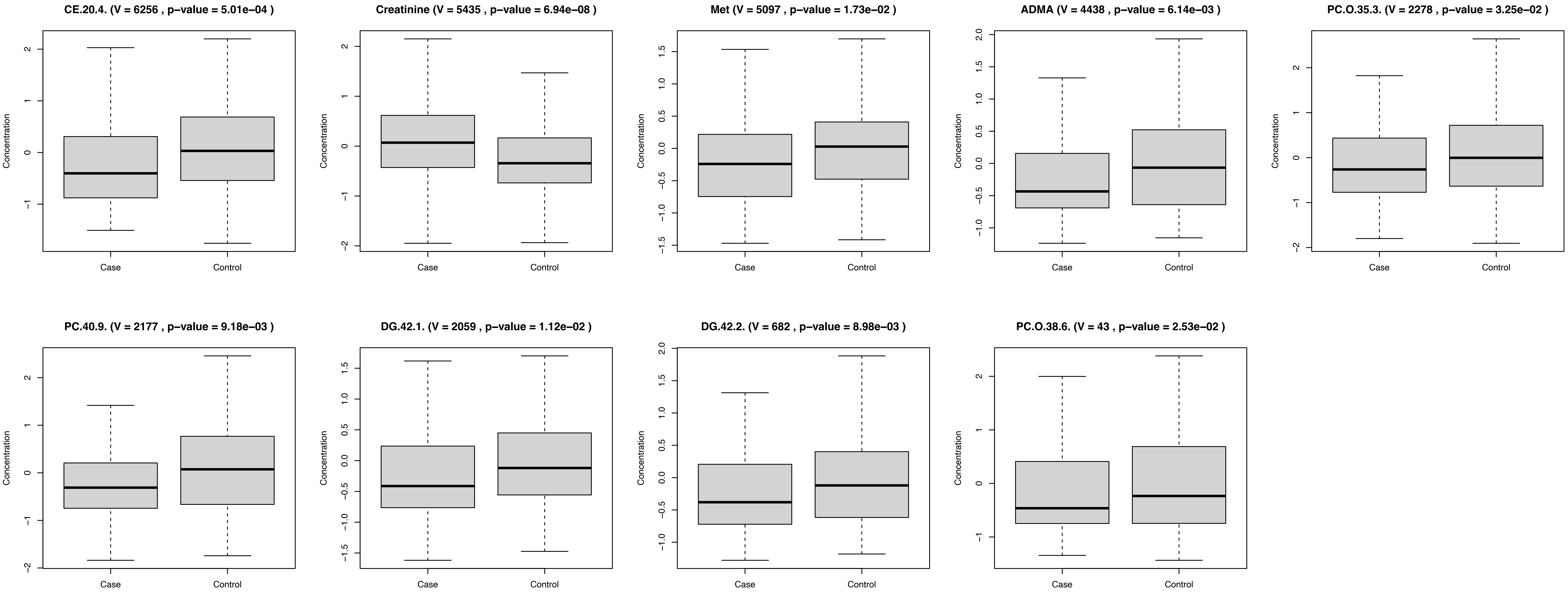

Supplementary Data 1. Differentially abundant metabolites between carriers and non-carriers of the 22q11.2 deletion. A total of 84 metabolites were found to be differentially abundant between carriers and non-carriers of the 22q11.2 deletion using the Wilcoxon signed-rank test. Box plots including V-statistics (V value) and FDR adjusted p-values are shown.
